# Hardness of cubic solid solutions

**DOI:** 10.1038/srep40276

**Published:** 2017-01-05

**Authors:** Faming Gao

**Affiliations:** 1 Key Laboratory of Applied Chemistry, Yanshan Univesity, Qinhuangdao 066004, China

## Abstract

We demonstrate that a hardening rule exists in cubic solid solutions with various combinations of ionic, covalent and metallic bonding. It is revealed that the hardening stress ∆τFcg is determined by three factors: shear modulus *G*, the volume fraction of solute atoms *f*_*v*_, and the size misfit degree *δ*_*b*_. A simple hardening correlation in KCl-KBr solid-solution is proposed as ∆τFcg = 0.27 *G*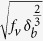. It is applied to calculate the hardening behavior of the Ag-Au, KCl-KBr, InP-GaP, TiN-TiC, HfN-HfC, TiC-NbC and ZrC-NbC solid-solution systems. The composition dependence of hardness is elucidated quantitatively. The BN-BP solid-solution system is quantitatively predicted. We find a hardening plateau region around the *x* = 0.55–0.85 in BN_*x*_P_1−*x*_, where BN_*x*_P_1−*x*_ solid solutions are far harder than cubic BN. Because the prediction is quantitative, it sets the stage for a broad range of applications.

High-hardness materials are widely used for wear-resistant coatings and cutting tools. In engineering practice, hardening processes, such as Hall-Petch strengthening, work hardening, precipitation hardening, and alloy hardening, are commonly used to increase the hardness of a material^1^. Solid-solution hardening is a type of alloying that is performed by adding a soluble alloying element to the material to be hardened. Such mixtures often exhibit superior tunable properties compared to those of pure materials. Obviously, a physical understanding of the hardening mechanisms of solid solutions is critical for the development of novel wear-resistant materials. Although solid-solution hardening in metals has been extensively studied^1^,^2^,^3^,^4^, we are currently far from achieving a quantitative understanding of the hardening behavior of solid solutions.

In recent years, computational materials science based on first-principles calculations has emerged as an effective supplement to experimentation, especially because it enables powerful prediction of material properties that depend only on electronic density or atomic bonding. Because hardness is the resistance to localized fracture or permanent plastic deformation, it relies strongly on the motion of dislocations in addition to atom bonding^1^,^5^. Technically, both dislocations and solid solutions represent great challenges for the current first-principles calculations. Therefore, solid-solution hardening cannot be predicted strictly from the electronic structure obtainable using density functional theory calculations. From a electronic transition viewpoint, the hardness formulas developed by Gao *et al*.^5^ and other scientists^6^,^7^,^8^,^9^ are very successful for perfect elemental or binary crystals without any defects and solid solute. However, these schemes encounter insurmountable difficulty in reproducing correctly the trend of the hardness increase with composition in solid solutions. Recently, Gilman^2^ tried to explain the hardening of Ag-Au and KCl-KBr solid solutions using mixing heat, strain and mixing entropy, respectively. From our initial theory^5^, we learned that the activation energies of dislocation glide and hardness are proportional to homopolar band gap *E*_h_. When normal sites are replaced by other atoms or vacancies in a crystal structure, the local electronic states or new energy levers are introduced, leading to a 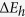. When 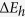 is positive, the activation energies of dislocation glide or hardness will increase. Therefore, a hardening rule could exist in various solid solutions with combinations of ionic, covalent and metallic bonding. However, an accurate expression of 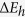 can not yet be extracted directly from the electronic description of impurity-dislocation interactions. According to Fleischer’s continuum elasticity scheme^1^, when solute and solvent atoms in solid solutions differ in size, local stress fields are formed on a slip plane, and these stress fields interact with those of the dislocations, impeding their motion. As a result, to move the dislocations higher applied stress is required, leading to the increase of material hardness. In the framework of the Fleischer-Friedel point-obstacle approximation^1^, the maximum interaction force *F*_m_ between a dislocation and a single solute atom is characterized by a critical breaking angle *θ*_c_: *F*_m_ = 2*E*_d_sin*θ*_c_, where *E*_d_ is the dislocation line energy. Furthermore, the critical stress of solid solutions can be estimated by 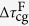 = 1.8*Gδ*_*b*_ *f*^1/2^, where *G* is shear modulus, *f* is the volume fraction of solute atoms intersecting the unit area of the glide plane, in the isotropic case the size misfit parameter *δ*_*b*_  = (*da*/*dc*)/*a, a* is the lattice constant, *c* is the atomic solute concentration^1^.

From the perspective of dislocation, the hardness *H*_k_ of a single-crystal material relies strongly on the resolved shear stress on the glide plane due to indentation. We proposed a hardness formula. The Knoop hardness of (*hkl*)[*h’k’l’*] direction in single crystals can be expressed as follows^10^:


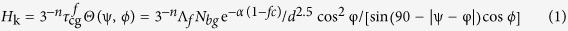


where 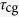 is the stress required for slip by the movement of lattice planes past one another in the glide region, *Θ*(ψ, ϕ) is the orientation factor of a glide plane, ϕ is the angle between a glide-plane spacing vector and the section of indenter along the short edge, and ψ is the angle between the projection of a glide-plane spacing vector on the section of indenter along the short edge and the short edge of the indenter, φ is the angle between the facet and the crystal surface, Λ_*f*_ is a constant, *N*_*bg*_ is the bond electron density in the *glide region, f*_c_ is covalency, *d* is bond length. Parameter *n* is taken as 0 and 1 for brittle and semi-brittle materials, respectively. In solid-solution single crystals, the total hardness *H*_k_ of solid-solution single crystals can be expressed as follows:


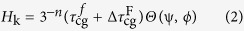


The hardening stress 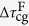 is determined by three factors: shear modulus *G*, the volume fraction of solute atoms *f*_*v*_, and the size misfit degree *δ*_*b*_. 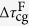 may be expressed in a general form


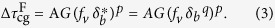



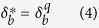


where A, *p, q* are constants, 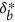 is termed “effective atomic size misfit”.

The most of alkali halides and metals are semi-brittle materials. The solubilities for the alkali halide solid solution are often limited, but some are complete. In the KCl-KBr system, the two compounds are entirely soluble in each other. Thus, it provide a very suitable model system for studying details of hardening of solid solutions. Some data points for the system were determined by Subba *et al*.^11^. We derive the fitting formula for the hardening stress 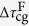 from KBr_*x*_Cl_1−*x*_ solid solution:


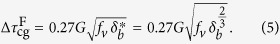


where we take *f*_*v*_  = 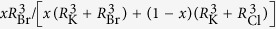, (*x* < 0.5). Figure 1 shows the good agreement between theoretical and experimental hardness values of KBr_x_Cl_1−x_ solid solution. Where the values of shear modulus and the lattice parameters are obtained via linear interpolation of the values for the end members. The Ag-Au alloy also is a semi-brittle material. And it is a classical example of the existence of a entire series of fcc solid solutions. Both bulk and film samples are single-phase alloys for any x^12^. We calculate hardness values of Ag, Au and Ag-Au alloy using Eq. 5, which are listed in Table 1. From Fig. 2, itcan be seen that the calculated hardening amplitude of Ag-Au alloy is in agreement with experimental results of bulk and film samples.

In addition to semi-brittle materials, a nonlinear increase of microhardness vs composition was observed on brittle solid solution single crystals of the InP-GaP. However, the hardening degrees are not significant compared to those of semi-brittle materials abovementioned. For semi-brittle materials the dislocations are mobile at a slip plane, In brittle materials the dislocations do not move at room temperatures, they tend to fracture along a slip plane before yield can occur. Equation 1 reveals a correlation of the critical shear stress for slip between semi-brittle and brittle materials by index *n*. Therefore, we may employ Eqs 2 and 5 (take *n* = 0, p = 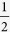, q = 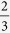) to estimate the hardening stresses in InP-GaP brittle solid solution. The good agreement between experimantal and calculated results can be seen from Fig. 3.

Transition-metal carbides and nitrides are important bulk and thin-film materials and widely used for cutting tools and wear-resistant coatings^15^,^16^. Due to the complexity of strong d-electron effect the hardness of transition-metal carbides and nitrides solid solutions is not yet completely understood at a fundamental level. Jhi *et al*.^17^ attempted uncovering veil for the solid solution hardening, but studied only the effect of valence electron concentration (VEC) on shear modulus. Hu *et al*.^18^ noted that shear modulus cannot be used as a rigorous measure of the hardness of covalent/ionic crystal solid solutions. Hugosson *et al*.^19^ proposed a mechanism to enhance hardness in *multilayer* transition metal carbide/nitride coatings. Such type of hardening is attributed to a large number of different glide-systems suppressing the propagation of dislocations, which is consistent with the theory of hardness^10^. However, they did not give any quantitative method for the hardness increases. In the transition-metal carbide and nitride solid solutions, the hardness enhances may attributed to solute atoms hindering dislocation movement. The predicted hardness enhancements of transition-metal carbide and nitride solid solutions by Eq. 5 are shown in Fig. 4. For comparison, Hu’s calculated results are also displayed. From Fig. 4a, it is seen that the deviation between the calculated hardness by Hu *et al*. and the corresponding experimental values are great although they try to reveal composition dependence of hardness of TiN_x_C_1−x_. In contrast, our scheme reproduce the hardness trend with x correctly. It should be noted that the deviation still exist using Eq. 5. The carbides and nitrigencarbides of the transition metals Ti, Zr, Hf and Nb all have the NaCl structure, which consists of close-packed planes of metal atoms. The C and N atoms locate in the octahedral holes. Their bonding is a combined covalent-metallic-ionic type of chemical bond, which is far more complex than that of alkali halides, Au-Ag and InP-GaP solid solutions. Therefore, for transition metal nitrigencarbides the parameters (*A, p, q*) in Eq. 5 might need to adjusted appropriately.

The search for novel hard materials would benefit from the advance in the theoretical treatment of solid solutions. We take BN-BP solid solutions as example. The hardness of binary BN-BP solid solutions depends strongly on their composition. Figure 5 shows the results for the BN-BP system. In particular, a ultrahigh hardness plateau region around the *x* = 0.55–0.85 in BN_*x*_P_1−*x*_ is predicted. As evident in Fig. 5, BN_0.75_P_0.25_ (B_4_N_3_P) are hardest among BN-BP solid solutions. Its hardness value, 61 GPa is far greater than that of cubic BN, 46 GPa. Thus, it is a candidate for new superhard materials. To confirm structure and structural stability of BN_0.75_P_0.25_ (B_4_N_3_P), we performed first-principles calculations using DFT code and the Vienna Ab-initio Simulation Package (VASP)^20^,^21^,^22^,^23^,^24^. The calculated results show that B_4_N_3_P exhibit a zinc-blende structure. The calculated lattice parameters of cubic B_4_N_3_P were 3.896 Å. The calculated elastic constants for B_4_N_3_P were C_11_ = C_22_ = C_33_ = 565 GPa, C_44_ = 297 GPa, and C_12_ = 132 GPa. The mechanical stability criteria in a cubic crystal are given by C_11_ > 0, C_44_ > 0, C_11_ > |C_12_|, (C_11_ + 2C_12_) > 0. The elastic constants of B_4_N_3_P clearly satisfy all of the mechanical stability criteria, indicating their elastic stability. The calculated bulk modulus and shear modulus values for B_4_N_3_P were 339 GPa and 265 GPa, respectively. The phonon dispersion curves of B_4_N_3_P at ambient pressure are presented in Fig. 6. No imaginary frequencies were observed throughout the whole BZ, confirming the dynamic stability of the diamond-structured cubic phases. Moreover, the calculated electronic band structures of B_4_N_3_P showed that the band gap is 1.7 eV under ambient conditions, indicating a good semiconductivity. Using modern high-pressure–high-temperature technique, the B_4_N_3_P could be fabricated if a suitable synthesis route could be developed.

In conclusion, we demonstrated the origin of hardening of cubic solid solutions. A hardness formula was developed. The theoretical prediction for BN-BP solid solution indicated that the B_4_N_3_P solid solution single crystal possesses a superhardness that exceeds that of cubic BN. The successful application of this method would contribute to the continuing search for novel hard solid solutions for industrial applications.

## Methods

The total energies are calculated by using a plane-wave pseudpotential method based on density functional theory (DFT), implemented in Vienna Ab initio Simulation Package (VASP)^20^,^21^. The general gradient approximation (GGA) of the Perdew-Burke-Ernzerhof parameterization was used for the exchange and correlation functions^22^. Integrations over the Brillouin zone (BZ) were performed using Monkhorst-Pack (a tetrahedron method for BZ integration) grids^23^. Optimization of the structural parameters was achieved by the minimization of forces and stress tensors. Calculations of the phonon dispersions were carried out using the density functional perturbation theory (DFPT) within the local density approximation in a plane-wave basis, as implemented in the Quantum ESPRESSO code with the Troullier–Martins (TM) pseudopotentials^24^. The elastic constants were determined by applying an appropriate set of distortions with the distortion parameter varying between −0.02 and +0.02. In K_Br_, the suggested radius of the Br atom is *R*_Br_ = *r*_Br_ *d*/(*r*_K_ + *r*_Br_)*f*_i_ + *f*_c_ *d*/2, where *r*_Br_ is the effective ionic radius of the Br atom, *d* is the bond length, and *f*_i_ and *f*_c_ are the ionicity and covalency^10^, respectively.

## Additional Information

**How to cite this article**: Gao, F. Hardness of cubic solid solutions. *Sci. Rep.* **7**, 40276; doi: 10.1038/srep40276 (2017).

**Publisher's note:** Springer Nature remains neutral with regard to jurisdictional claims in published maps and institutional affiliations.

## Figures and Tables

**Figure 1:**
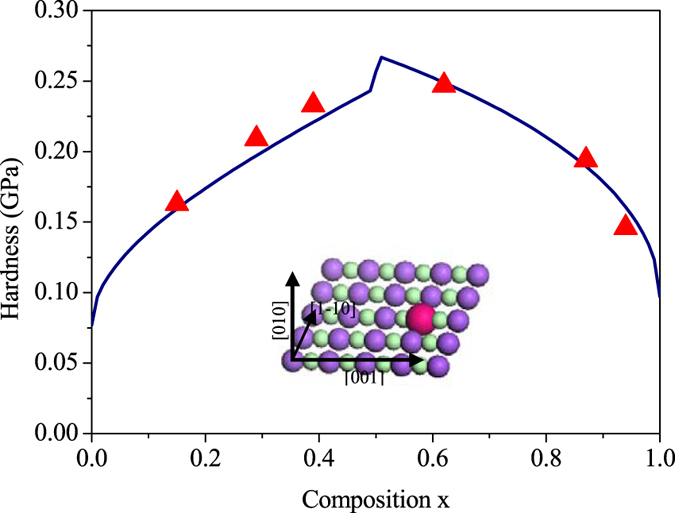
Experimental and calculated hardness enhancement on (100) planes plotted against composition *x* for KBr_*x*_Cl_1−*x*_ solid-solution systems. The triangles indicate experimental data (from ref. 11). The blue curve represent the calculated data. The inset is a schematic of the (110) slip plane, where the red ball is the solute atom and the other balls are solvent atoms.

**Figure 2:**
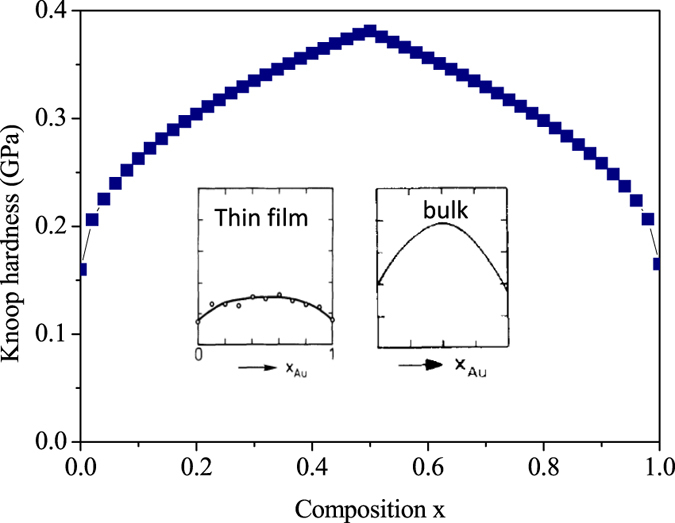
Calculated hardness on (100) planes plotted against composition x for the Ag-Au solid solutions. Inset, microhardness of bulk alloys (right) and thin films (left) samples (from ref. 12).

**Figure 3:**
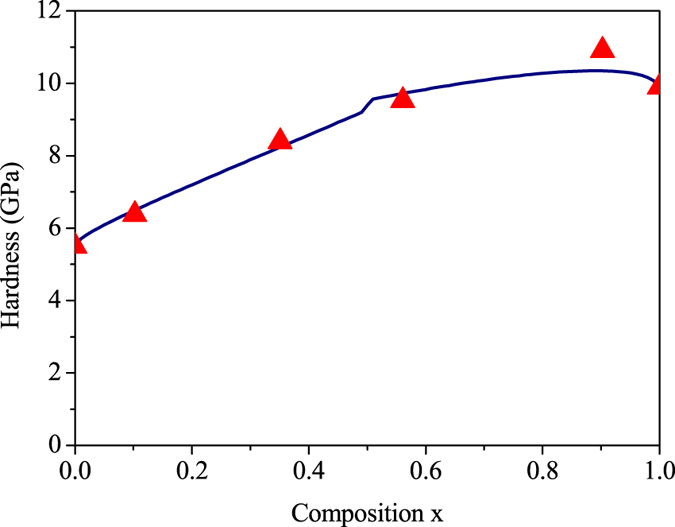
Experimental and calculated hardness enhancement on (100) planes plotted against composition *x* for Ga_*x*_In_1−*x*_P solid-solution systems. The red triangles indicate experimental data (from ref. 14). The blue curve represent the calculated data.

**Figure 4:**
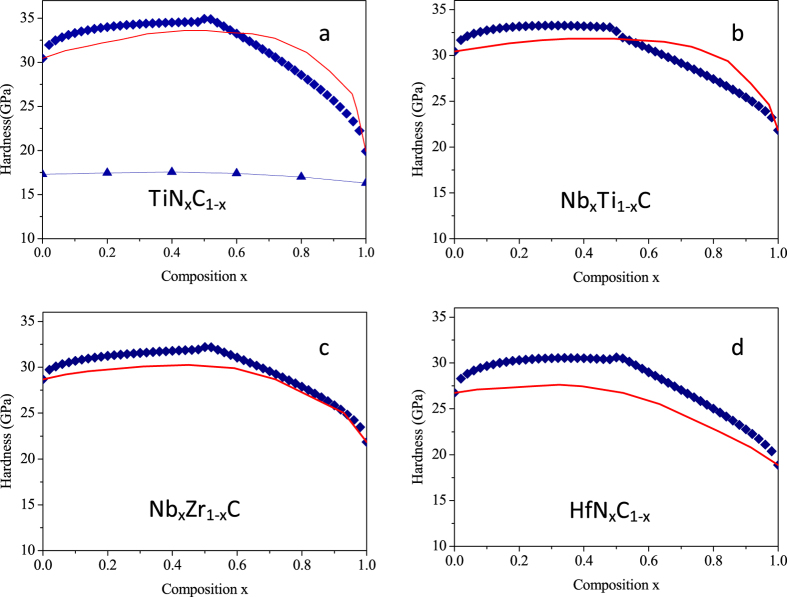
Experimental and calculated hardness enhancement on (100) planes plotted against composition *x* for various solid-solution systems. The red curves indicate experimental data (from ref. 16). The blue diamond represent the calculated data by this work. The blue triangle represent the calculated data from ref. 18.

**Figure 5:**
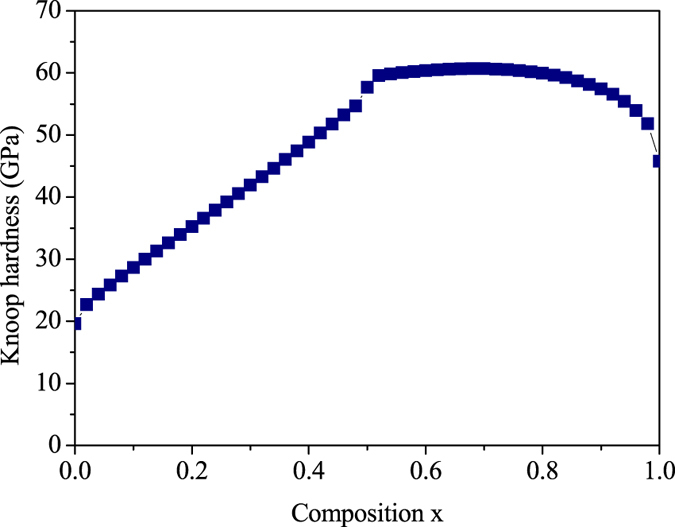
Calculated Knoop hardness on (100) planes plotted against composition *x* for the BN_*x*_P_1−*x*_ solid solutions.

**Figure 6:**
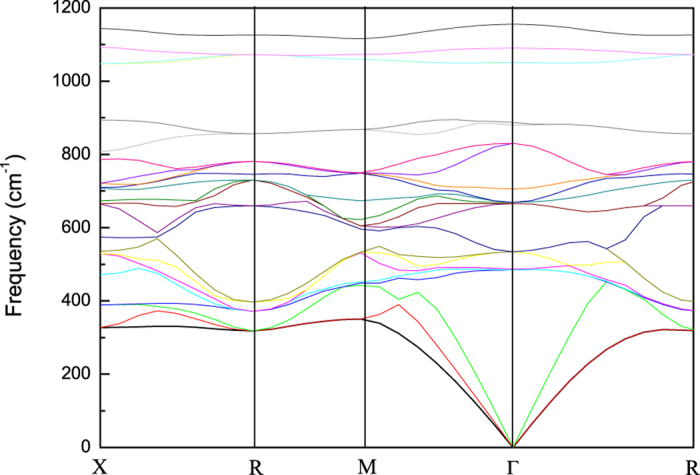
Theoretical phonon band structures of B_4_N_3_P at ambient pressure.

**Table 1:** Calculated Knoop hardness.

Crystal	Slip system	Indent diagonal orientation	ψ (°)	ϕ (°)	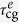 (GPa)	*H*_*k calc*._ (GPa)	*H*_*v expt*._ (GPa)	*G* (GPa)	*δ*_*b*_ (10^−3^)	*f*_v_	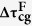 (GPa)
Ag	(111)[1 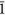 0]	(100)[110]	35.264	0	0.172	0.14	0.251	30.3			
		(100)[001]	45	35.264	0.172	0.18					
Au	(111)[1 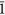 0]	(100)[110]	35.264	0	0.173	0.14	0.216	27.5			
		(100)[001]	45	35.264	0.173	0.19					
Cu	(111)[1 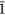 0]	(100)[110]	35.264	0	0.337	0.28	0.369	44.7			
		(100)[001]	45	35.264	0.337	0.36					
Au_0.5_Ag_0.5_									1.96	0.50	0.23

For comparision, experimental Vickers hardness H_v expt._ (from ref. 13) are listed.
